# Design and pilot evaluation of a brief intervention to reduce transphobia and improve attitudes of government officials to address legal gender affirmation needs of transgender people in Peru

**DOI:** 10.1080/16549716.2024.2381881

**Published:** 2024-07-22

**Authors:** Sari L. Reisner, Amaya Perez-Brumer, Ximena Salazar, Alfonso Silva-Santisteban

**Affiliations:** a Department of Epidemiology, University of Michigan School of Public Health, Ann Arbor, MI, USA; b Department of Epidemiology, Harvard T.H. Chan School of Public Health, Boston, MA, USA; c The Fenway Institute, Fenway Health, Boston, MA, USA; d Division of Social and Behavioural Health Sciences, University of Toronto, Toronto, Canada; e Center for Interdisciplinary Research in Sexuality, AIDS and Society, University of Peru Cayetano, Lima, Peru

**Keywords:** Transgender, legal epidemiology, global health, program development, health disparities

## Abstract

Legal gender affirmation – legal name and gender marker change – is an important health-promoting health determinant for transgender people. In Peru, the state's failure to universally recognize transgender people’s legal identity limits standardized legal affirmation procedures, including the paucity of government officials trained in gender affirmation strategies. This project, in partnership with Registro Nacional de Identificación y Estado Civil (RENIEC) and transgender communities, designed and piloted a group-based intervention to sensitize government officers to the importance of gender-concordant identity documents. Between August 2017 and February 2018, three in-person group intervention sessions were held (each 3–4 hours) with 51 government officers. Guided by Gender Affirmation and Structural Violence Frameworks, the intervention utilized Adult Learning Theory and applied storytelling and testimonials as pedagogy. Pre-/post-test surveys were administered (19 true/false items, summed to create an index score measuring knowledge and attitudes toward transgender people). Within-person changes in pre-/post-intervention scores were evaluated using paired t-tests. Pre-/post-test data were available for 41 participants. After the intervention, there were improvements in knowledge and more favorable attitudes toward transgender people (pre-test mean = 14.09, SD = 2.33 vs. post-test mean = 15.62, SD = 1.82; difference = 1.53, 95% CL = 0.60, 2.67; t-test = 3.30 [df = 46]; *p* = 0.002). The intervention was feasible to conduct and garnered high acceptability. The results suggest the promise of this brief intervention for future research and testing before potential later implementation and scale-up to increase the capacity of government officers to address legal gender affirmation for transgender people in Peru.

## Paper Context

- **Main findings:** A brief group-based theoretically informed intervention designed and piloted by a multidisciplinary cross-sector team in partnership with transgender communities was feasible to conduct, garnered high levels of acceptability, and significantly increased knowledge and favorable attitudes toward transgender people for government officers responsible for identity documents in Peru.
- **Added knowledge:** Legal gender affirmation is an important determinant of mental health and wellbeing for transgender people; this evidence-based intervention increased the capacity of government officers to meet health-harming legal gender affirmation needs, specifically legal name and gender marker change, addressing a structural barrier to legal gender affirmation for transgender people in Peru.
- **Global health impact for policy and action:** Findings underscore the promise of this intervention for future research and testing before potential later implementation and scale-up in Peru, and for adaptation in other countries and contexts to address the training and capacity of government officials to effectively process and implement legal gender affirmation, a structural barrier to legal gender affirmation for transgender people.

## Background

Globally, transgender people experience myriad health inequities fueled by widespread social and economic marginalization and exclusion [1,2]. Legal gender affirmation – legal name and gender marker change (i.e. gender concordant identity documents) – is an important determinant of mental health and wellbeing for transgender people [3,4]. Legal gender affirmation has been associated with positive health outcomes among transgender people, including improved mental health (lower reports of serious psychological distress and global psychiatric distress) [4,5], substance use (lower rates of smoking) [6], and reported reduction in experiences of stigma and discrimination in the public health system, educational system, or with security forces [7]. Yet, in many global contexts, including in Peru, legal gender affirmation is impeded by structural barriers that create substantial and avoidable health-harming legal needs as well as unjustly make transgender people the targets of discrimination [8].

In Peru, significant barriers exist in accessing legal gender affirmation (legally changing their names and gender markers) of transgender individuals’ identities on government-issued identity documents. Peru's current legal framework does not offer a straightforward process for transgender people to update their names and gender markers [9]. Instead, they must navigate complex, inconsistent legal and administrative barriers. The procedures for legal gender affirmation are burdensome, costly, and complicated, often depending on the discretion of individual government officials, and may involve appealing their case [10]. The *Documento Nacional de Identidad* (DNI; National Identity Document) is a document that is essential for accessing healthcare, employment, banking, and participating in political processes like voting. While some transgender people in Peru can legally change their names and gender markers on identity documents irrespective of gender-affirming care access, the process of doing so does not uphold the standard recognized under international human rights norms [11,12]. For example, the human rights standard espoused in the global Yogyakarta Principles is that transgender people have the right to access a quick, transparent, and accessible mechanism to change gender and names under the right to self-determination.

In contrast, Argentina’s Gender Identity Law, enacted in 2012, allows transgender individuals to change their legal gender without requiring a medical diagnosis, gender-affirming surgery, or judicial approval. This law enables individuals to self-identify and have their gender identity accurately reflected on all official documents through a defined, albeit heterogeneous, administrative process [7,13]. In the absence of a national Gender Identity Law in Peru, the decision to approve a legal name or gender marker change occurs on a case-by-case basis, made by individual judges following legal processes. Furthermore, there is a paucity of government actors with cultural competency or training in transgender rights to process and implement legal affirmation cases effectively.

To our knowledge, no evidence-based interventions exist in Peru to change attitudes toward transgender people. Peruvian society is polarized in terms of attitudes toward transgender people and their legal rights. The Peruvian panel of the Global Attitudes Toward Transgender People Survey revealed that nearly half (48.5%) of respondents did not ‘strongly agree’ that transgender people should be protected from discrimination [14]. There is an urgent need for efforts to reduce negative attitudes toward transgender people, including those that reduce transphobia, for government actors.

This article describes the design and pilot evaluation of a theoretically informed intervention, with government officers overseeing legal name and gender marker change processes in Peru to address the structural barrier of lack of training and capacity to implement legal gender affirmation for transgender people. This project evaluated the intervention’s feasibility, acceptability, and preliminary impact on knowledge and attitudes toward transgender people. The goal of the intervention was to improve the capacity of government officers to implement, advocate, and address legal gender affirmation for transgender people more effectively.

## Methods

Between August 2017-February 2018, UNICXS, a multidisciplinary and cross-sectoral network formed to document and respond to human rights violations against transgender people in Peru, collaborated with the Peruvian National Identity Registry (Registro Nacional de Identificación y Estado Civil [RENIEC]) to improve the quality of services for transgender people requesting their identity documents or changes to them. A brief single-session group-based intervention to sensitize officers to improve the quality of services delivered to transgender people requesting their DNI was developed, piloted, and evaluated using a non-randomized pre-/post-test design. Three intervention group sessions were held (each lasting between 3–4 hours) with a total of 51 RENIEC officers. Each intervention session was delivered in-person at RENIEC’s premises. RENIEC collaborators oversaw logistics and participant recruitment. Two members of the UNICXS team delivered the intervention. As a program evaluation, the project was not human subject research.

### Intervention development and description

To inform the intervention, a needs assessment was conducted with transgender community partners, including testimonials of their experiences with DNI and legal name and gender marker change processes. A review of existing RENIEC protocols was also conducted to identify how to better serve transgender people and what sensitizing education was needed for government actors. The intervention was also informed by the team’s prior activities with RENIEC. As part of the collaboration, training workshops were requested by RENIEC, and were co-developed and implemented by the UNICXS team.

#### Sensitizing frameworks

Two sensitizing frameworks guided intervention content (see Table 1). First, the Gender Affirmation Framework conceptualizes that the need to be recognized or affirmed in one’s gender, when thwarted, leads to adverse health for transgender people [15]. Further, multiple components of gender affirmation – social, psychological, medical, and legal – matter for health [16]. Second, the Structural Violence Framework describes violence perpetrated by social structures, government institutions, and other institutionalized forms of violence as that which denies or prevents transgender people from meeting their basic needs and fully engaging in society [17].

**Table 1.** Overview of intervention content.

Topic #	Topic	Description	Sensitizing Framework	Time Allocation
1	Sex, gender, and sexuality	General sensitization on concepts, scientific consensus, biopsychosocial perspective on sex, gender, and sexuality and their interrelationships	Gender affirmation	15 min
2	Gender identity	Definition, gender norms, general attitudes and knowledge about transgender identity	Gender affirmation	15 min
3	Social situation of transgender women and men in Peru	Social needs, access to basic rights, limited protective legal framework, barriers to access public services including identity documents, unmet health-harming legal needs	Structural violence	15 min
4	Discrimination and violence	Everyday discrimination, direct violence, abuse of authority	Structural violence	15 min
5	Facilitated discussion	Q&A and discussion prompts	–	30 min
6	Testimonials	Transgender community members giving testimonials about lived experiences with DNI and legal gender affirmation needs	Gender affirmation	60 min
7	Facilitated discussion	Q&A and discussion prompts	–	30 min

#### Pedagogy

Adult Learning Theory was applied through facilitated discussions that were oriented toward problem-solving challenges that might arise in serving transgender people, rather than content-oriented materials, knowing that adults are most interested in learning material with immediate relevance and impact on their job [18]. Storytelling and testimonials were used as a pedagogical device to connect transgender communities’ lived experiences with participants’ learning to achieve desired learning objectives [19].

#### Content and duration

The brief single session intervention was designed to be 3–4 hours and delivered in-person. Transgender people’s lived experiences directly informed and shaped the content and delivery of the intervention, which included: General sensitization about sex, gender, and sexuality; gender identity; the social situation of transgender women and men in Peru; and discrimination and violence (see Table 1). The methodology consisted of didactic slide presentations, group discussions, and participation facilitated by presenters through targeted prompts and transgender community member testimonials.

### Intervention evaluation

Feasibility was evaluated as the proportion of participants who completed the intervention and the proportion who completed the pre-/post-test survey. Acceptability information was gathered verbally from participants who attended the groups and descriptively documented in field notes of intervention facilitators.

Pre- and post-test surveys were fielded to assess knowledge about transgender identity, legal protection, and attitudes towards transgender people. Nineteen true/false survey items were developed in consultation with RENEIC, transgender communities, and the multidisciplinary US–Peru public health research team and/or or adapted based on a prior national study of attitudes toward transgender people in Peru [14] (see Table 2). The 19 items were summed up to create an index (range 0–19). Surveys were completed using pen and paper immediately before and after each group intervention session. No other information was collected from intervention participants.

**Table 2.** Pre- and Post-Test Survey Items in Spanish and English.

#	Spanish Language: Marca si es verdadero o falso:	English Language: Mark true or false:	Correct Answer	Pre-Test		Post-Test	
	La discriminación hacia las personas trans se manifiesta … .	Discrimination towards trans people manifests … .		n	%	n	%
Q1	Solo a través de la violencia verbal	Only through verbal violence	F	41	87.23	45	95.74
Q2	En aquellos sectores con menos niveles de educación y acceso a la información	In those sectors with lower levels of education and access to information	F	39	82.98	35	74.47
Q3	De múltiples formas como violencia, física, psicológica, verbal, estructural	In multiple forms like violence, physical, psychological, verbal, and structural	T	43	91.49	46	97.87
	**Marca si es verdadero o falso:**	**Mark true or false:**					
Q4	Ser trans es una enfermedad	Being trans is a disease	F	47	100.00	47	100.00
Q5	Las mujeres trans son homosexuales	Trans women are homosexual	F	35	74.47	41	87.23
Q6	Las personas trans pertenecen a una población vulnerable	Trans people belong to a vulnerable population	T	31	65.96	36	76.60
Q7	La identidad de género de una persona puede corresponder o no con el sexo que se le atribuyó al nacer	Gender identity of a person can correspond or not with the sex that was attributed to them at birth	T	32	68.09	39	82.98
	**Marca si es verdadero o falso:**	**Mark true or false:**					
Q8	Burlarse de alguien no es violencia	Making fun of someone is not violence	F	45	95.74	41	87.23
Q9	La información ayuda a disminuir Ia discriminación hacia las personas trans	Information helps to decrease the discrimination towards trans people	T	46	97.87	45	95.74
Q10	A veces las personas trans se merecen Ia violencia que reciben	Sometimes transgender people deserve the violence they receive	F	45	95.74	45	95.74
Q11	Los estereotipos son Ia base de la discriminación	Stereotypes are the basis of discrimination	T	32	68.09	37	78.72
	**Marca si es verdadero o falso:**	**Mark true or false:**					
Q12	El Perú tiene protocolos y procedimientos para proteger a las personas Trans	Peru has protocols and procedures to protect Trans people	F	40	85.11	41	87.23
Q13	La ley de ldentidad de Género permitirá cambiar el nombre en el DNI	Gender Identity Law will allow people to change their name on their ID	T	18	38.30	31	65.96
Q14	Las personas trans tienen derecho a cambiar su nombre y sexo en el DNI	Trans people have the right to change their name on their ID	T	24	51.06	37	36.17
Q15	Las personas trans deben ser llamadas por su nombre del DNI para cualquier trámite en dependencias públicas	Trans people should be called by their name on IDs for any procedure in public dependencies	F	10	21.28	17	36.17
	**Tener información sobre las personas trans … .**	**Having information about trans people … .**					
Q16	Me ayuda a realizar mejor mi trabajo y mejorar el vínculo de la institución con una población vulnerable	Helps me to do a better job and improve the link of the institution with a vulnerable population	T	33	70.21	40	85.11
Q17	No es relevante porque nosotros tenemos que respetar a todas las personas por igual sean trans o no	Is not relevant because we have to respect all people the same if they are trans or not	F	18	38.30	20	42.55
Q18	No es relevante porque yo solamente sigo órdenes institucionales	It is not relevant because I only follow institutional orders	F	43	91.49	45	95.74
Q19	Me ayuda a conocer y comprender más a las personas trans para hacer mejor mi trabajo y atender bien a esta población	It helps me to know and understand more about trans people to do a better job and serve this population well	T	40	85.11	46	97.87

### Data analysis

For pilot-testing, descriptive statistics (frequency, proportion, mean, standard deviation) were summarized for index items and scores. First, differences in pre- and post-test scores for each group of participants (separately for groups 1–3) were evaluated using analysis of variance (ANOVA). No differences were observed across groups in pre-test scores (F-test value = 0.47 [df = 2], *p* = 0.626 or post-test scores (F-test value = 1.15; [df = 2]; *p* = 0.325). Overall mean scores across all groups were combined and compared in aggregate. Paired t-tests calculated differences in test scores pre- vs post- delivery of the intervention to compare within-person change. Analyses were conducted in SAS v9.1.

## Results

### Feasibility and acceptability

Of the 51 participants, all completed the intervention: Group 1 (*n* = 16), Group 2 (*n* = 20), and Group 3 (*n* = 15). Pre- and post-test data were available for 41 participants (92.16%). Group 1 had 4 participants who had to leave the session promptly and were unable to complete a post-test evaluation. The intervention had high levels of acceptability, with participants reporting the value of the information provided on sexuality and gender. They also appreciated the opportunity to listen to the experiences of transgender people and understand first-hand the importance of gender identity recognition in their lives.

### Pre-/Post-test assessment

Table 2 presents the frequency of each individual survey item from the pre-/post-test surveys. Table 3 displays the mean scores before and after intervention. There was a within-person increase in pre-test and post-test mean scores (pre-test = 14.09, SD = 2.33 vs post-test = 15.62, SD = 1.82; overall mean difference = 1.53, 95% CL = 0.60, 2.67) suggesting improvements in knowledge and more favorable attitudes toward transgender people after the intervention (t-test value = 3.30 [df = 46]; *p* = 0.002).

**Table 3.** Intervention pre- and post-test mean scores among government officers receiving the intervention (*n* = 47).

	Pre-Test Score	Post-Test Score	Mean Difference Pre-/Post-Test Score		
	Mean (SD)	Mean (SD)	Mean (95% CL)	t-test (df)	*p*-value
**All Groups (*n* = 47)**	**14.09 (2.33)**	**15.62 (1.82)**	**1.53 (0.60, 2.67)**	**3.30 (46)**	**0.002**
Group 1 - Training on 8/28/2017 (*n* = 12)	14.08 (2.11)	16.00 (0.95)	1.92 (0.26, 3.57)	2.54 (11)	0.027
Group 2 - Training on 1/31/2018 (*n* = 20)	13.75 (1.86)	15.15 (2.11)	1.40 (0.14, 2.67)	2.32 (19)	0.032
Group 3 - Training on 2/7/2018 (*n* = 15)	14.53 (3.04)	15.93 (1.91)	1.40 (−0.94, 3.74)	1.28 (14)	0.220

Paired t-tests calculated within-person change in mean test scores pre- vs post- delivery of the intervention. SD = Standard Deviation. df = degrees of freedom. Bolded row indicates the preliminary impact of the intervention across all three groups.

## Discussion

Applying Gender Affirmation and Structural Violence Frameworks [15–17], this theoretically informed intervention leveraged a cross-sector academic-governmental and transgender community partnership aimed to create, implement, and evaluate a sensitization intervention for Peruvian government officers responsible for identity documents to advance gender-affirming practice. The pilot evaluation suggested that the intervention was feasible to conduct, garnered high acceptability from participants, and was associated with improvements in knowledge and more favorable attitudes toward transgender people comparing pre- and post-test assessments. In the absence of Peruvian state-level gender identity legislation mandating universal access to legal name and gender marker change [10,20] in accordance with the standard recognized under international human rights norms [11,12], our findings suggest the timely promise of this brief intervention to increase the capacity of Peruvian government officials to more effectively address legal gender affirmation and potential for adaptation to other countries and global contexts. Future research and testing are needed before potential later implementation and scale-up.

Several limitations warrant consideration. First, this was a non-randomized pilot design; thus, findings on the preliminary impact of the intervention are observational and may be subject to confounding. Second, pre-/post-test surveys did not collect demographic data; thus, no information about participant characteristics could be statistically adjusted in pilot evaluation analyses. Third, regarding the pre-/post-test assessment, several items demonstrated small decreases in knowledge, such as question 8 (‘making fun of someone is not violence’). This finding could partially be due to measurement error, while some survey items were drawn from a national survey of transgender attitudes in Peru [14], none were psychometrically validated measures. Alternatively, at baseline, government officers may have legally/administratively interpreted these items but were sensitized to the sociological meanings of these items through intervention. Limitations notwithstanding, this project developed and piloted a promising brief group-based intervention to increase the capacity of government officers to address the health-harming need for legal gender affirmation in Peru. We collected pilot data that can inform future research and testing before potential later roll-out and scale-up of the intervention in Peru. The findings may also be applicable to many kinds of legal environments, not just in Peru or countries with similar legal standards, since the training focuses broadly on the lives and human rights of transgender people.

## Conclusions

Interventions to address legal gender affirmation, a structural determinant of mental health and well-being for transgender people are urgently needed [3]. To effectively advance legal gender affirmation and address the unmet health-harming legal needs of transgender people, such as legal name and gender marker change, evidence-based interventions to train and build the capacity of government officials are required. Our findings underscore the promise of this brief intervention for future research and testing before potential later implementation and scale-up in Peru. Adaptation of the intervention for other countries and contexts is recommended to increase the capacity of government officers to address the legal needs of transgender people.
